# COVID-19 pandemic during the war in Tigray, Northern Ethiopia: a sequential mixed-methods approach

**DOI:** 10.3389/fpubh.2025.1553452

**Published:** 2025-04-16

**Authors:** Kibrom Teklay Gebru, Hailay Gebretnsae, Harnet Adane, Negasi Gebremeskel, Mulat Tadesse, Tsegay Hadgu, Brhane Ayele, Cinzia Destefanis, Maria Teresa Giraudo, Fulvio Ricceri

**Affiliations:** ^1^Centre for Biostatistics, Epidemiology, and Public Health, Department of Clinical and Biological Sciences, University of Turin, Orbassano, Italy; ^2^Tigray Health Research Institute, Tigray, Ethiopia; ^3^Tigray Regional Health Bureau, Tigray, Ethiopia

**Keywords:** COVID-19, war, pandemic, health system, challenge, Tigray

## Abstract

**Introduction:**

The overlapping global crises of war, pandemic, and inflation have hit the poorest countries the hardest. Political and security risks have risen in nearly all nations, with those lacking resources suffering from significant preparedness gaps. Similar to other developing regions, Ethiopia’s Tigray has experienced many of these effects. This study analyzes the only available data to assess COVID-19 incidence and mortality trends and identify the key influencing factors.

**Methods:**

A quantitative analysis was performed using the available incidence and mortality data from the Tigray region prior to the war and from Mekelle town for the 11 weeks during the war. This analysis was complemented by qualitative insights obtained conducted through interviews. Vaccination data covering the years 2021–2023 were also available for the Tigray region. Multiple datasets were selected for comparison purposes based on their relevance to the research objectives. Key informant interviews were conducted with members of the regional response team. Narrative analysis and a deductive approach were applied for systematic coding and thematic analysis of the interviews using Atlas.ti. The study was conducted from 1 January 2023 to 10 December 2024.

**Results:**

The region established an Emergency Operation Center with seven pillars to coordinate the overall response efforts. The COVID-19 positivity rate before the war varied between 0.97 and 20%. During the war, when services were briefly resumed for 10 weeks in Mekelle city, 3,802 cases were detected from 13,213 tests conducted, resulting in a positivity rate of 28.8%. During the same period, 85 deaths were reported. Only 45.9% of the eligible population was fully vaccinated, while nearly 29% had received only the first dose of a two-dose vaccine. Despite challenges, key opportunities included government commitment, strong communication between local experts and international institutions, and local resource mobilization. However, the crisis exposed the fragility of the health system, leading to a significant loss of life.

**Conclusion:**

While the region initially managed to delay the onset of COVID-19 and maintain lower positivity and fatality rates, the war in Tigray led to severe disruption and the eventual collapse of the health system. As a result, the disease was largely neglected and deprioritized, even after the signing of the cessation of hostilities agreement.

## Background

The COVID-19 pandemic originated with the introduction of the novel coronavirus, SARS-CoV-2, in human populations on 31 December 2019 (1). COVID-19 was declared a pandemic on 11 March 2020. Public Health and Social Measures (PHSM) were implemented worldwide since the beginning of the pandemic to suppress SARS-CoV-2 transmission, reduce morbidity and mortality from COVID-19, and prevent overburdening of health systems and other critical societal functions (2, 3). Responses to COVID-19 present an opportunity to protect the lives, dignity, and well-being of individuals everywhere. Public health measures are most effective when the community participates in decision-making processes and agrees with the measures introduced (4). In addition to these measures, medical countermeasures such as the administration of drugs or vaccination are also essential to reduce morbidity and mortality from COVID-19 (3).

Globally, 777,026,543 confirmed COVID-19 cases and 7,078,481 deaths were reported to the WHO from 31 December 2019 to 8 December 2024 (5). Various mitigation measures are being put in place, through great efforts and at great cost, to address the impact of COVID-19 and reduce the risk of future crises, especially for the poorest and most vulnerable individuals and countries (6). However, limited skilled manpower, inadequate medical equipment, insufficient preparation, less prepared and weak health systems, low funding, and limited resources (i.e., limited supply of ventilators, electricity, and oxygen), poverty, and overcrowding increase the vulnerability of some population segments. They are among the challenges faced in most of the developing countries, including Ethiopia (7–9).

The COVID-19 pandemic has led to a dramatic loss of human life worldwide and presents an unprecedented challenge to public health, food systems, and the workplace environment (10). Young people are particularly vulnerable to the disruptions the pandemic has caused, and many are now at risk of being left behind in education, economic opportunities, and health and wellbeing during a crucial stage of their life development (11). Moreover, there have been major effects on household and individual incomes and increasing unemployment (12). A study has also recommended the need for medium- and long-term planning to rebalance and reenergize the economy following this crisis (13). The Global Health Security Index has indicated that the public health and health system capacities must be coupled with policies and programs that enable all people to comply with public health recommendations. This is one of the clear lessons from the COVID-19 pandemic (14).

On the other hand, armed conflict between warring states and groups within states has been a major cause of poor health and mortality for most of human history (15). It has often been reported that an increasing frequency or intensity of armed conflict would seriously affect efforts to cope with the pandemic. In areas with political unrest, conflicts, and overall social instability, setting up humanitarian programs is dangerous and requires complex logistics and strong negotiation skills between the parties involved. Besides, the low-income developing countries experienced significant declines in per capita income during the pandemic (7, 16, 17).

Similar to many countries, Ethiopia has been seeking to tackle the COVID-19 pandemic by implementing various strategies, including mass vaccination with second and booster doses. Ethiopia reported its first COVID-19 case on 13th March 2020. According to the WHO Coronavirus (COVID-19) Dashboard, by the end of 10th September 2023, a total of 500,872 confirmed cases of COVID-19 and 7,574 deaths had been reported by Ethiopia (5). It is also believed that the intensification of the conflict and instability, particularly in the North of the country, has drawn the attention of the authorities and the public away from the COVID-19 pandemic (18). The overlapping global crises of war, pandemic, and inflation are hitting the poorest countries hardest. Political and security risks have increased in nearly all countries, and those with the fewest resources have the highest risk and greatest preparedness gaps.

Tigray has been affected by the war that started on 4 November 2020. The Ethiopian National Defense Force and Amhara forces from Ethiopia, along with the Eritrean Defense Force from Eritrea, have been fighting against the Tigray Regional Forces and facing significant consequences. The region also faced El Niño-induced weather shocks that impacted rainfall, while a surge of desert locusts adversely affected agricultural production and economic conditions. Additionally, COVID-19 has compounded the effects of these factors in the region. Therefore, this study aims to analyze the available data to assess COVID-19 incidence and mortality trends and determine the key influencing factors in the Tigray region during the period, including the war.

## Methods

### Study setting

Tigray is one of Ethiopia’s 11 national and regional states, with a population of 7.3 million in 2021, based on the 2007 Ethiopian housing and population census located in the north. The region has seven administrative zones and 93 districts (57 rural and 36 urban). Tigray has long been affected by both natural and human-made disasters, including the 17-year-long civil war that ended in 1991. These disasters have negatively affected the health of the people in Tigray, with a pronounced effect on mothers and their children (17, 18).

Tigray’s health sector has been progressing across multiple dimensions over the last 25 years. Healthcare facilities in both the public and private sectors have expanded significantly, and the health workforce-to-population ratio is approaching global standards. Before the war, the region had more than 310 ambulances and a well-established referral system for primary health care—comprising 741 village health posts linked to 230 health centers and 24 primary hospitals. These facilities fed into the secondary level of care, which included 14 general hospitals in major towns, and further into the tertiary level, with two referral hospitals. The private health sector was also flourishing, with more than 750 private healthcare facilities, ranging from drug vendors and clinics to primary and general hospitals (19).

#### Study design and period

This research employs a mixed-sequential methods design, integrating both quantitative and qualitative data to provide a comprehensive description and comparison of the consequences of the deadliest war and the COVID-19 pandemic in Tigray from the declaration of the COVID-19 Public Health Emergency on 11th March 2020, up to 10th September 2023.

#### Data collection and sources

The study used an explanatory sequential mixed-methods approach, in which quantitative data were used to identify statistical patterns, and qualitative data were used to explore experiences, perspectives, and contextual factors influencing the data. First, quantitative data were collected from multiple datasets. The selection criteria included the availability of comprehensive data, the credibility and reliability of data sources, and their relevance to the research topic. The datasets were obtained from various reputable sources, such as government databases, international organizations, and academic and research institutions. These included databases from the Tigray Regional Health Bureau, the Ethiopian Public Health Institute, and the WHO. Subsequently, we conducted key informant interviews (KIIs) with purposively selected experts who were engaged in implementing activities to tackle COVID-19 in the Emergency Operation Center (EOC). These participants played a key role from the start of the pandemic until the end of the study period. The EOC for the pandemic had seven sub-pillars, and a total of 10 key informants participated. These informants were selected from different stakeholder groups that took the lead in the response to COVID-19. Experts from each pillar were also included in the survey.

Data extraction was performed systematically to ensure consistency and accuracy. Relevant variables and indicators were identified, data points for the specified time periods were extracted, and data integrity and completeness were ensured. Data were extracted from the databases and cleaned in Excel sheets.

### Data analysis

#### Quantitative part

Descriptive statistics were employed to summarize and describe the main features of the dataset. The results were visualized through graphical presentations such as figures and tables to enhance the interpretability of findings. Since data, whenever available, were limited to counts, it was not possible to use regression models that linked incidence to possible population factors.

#### Qualitative part

Qualitative data collected through key informant interviews (KIIs) were analyzed using Atlas.ti. A deductive approach was used for the systematic coding and thematic analysis of interviews, enabling the identification of key patterns, insights, and perspectives. The narrative analysis used qualitative data analysis (QDA) for in-depth context and a nuanced understanding of the findings, capturing rich, narrative details that might not be evident through quantitative methods alone. Insights from the qualitative interviews were used to explain or expand the statistical results, facilitating a deeper understanding of the relationships and trends identified in the numerical data.

#### Triangulation

The integration of qualitative and quantitative data strengthened the overall analysis by offering a mixed-methods approach. Triangulation was employed to ensure the reliability and validity of the findings. This involved cross-verifying data from multiple sources and using different data analysis methods. It helped reduce bias, enhance the credibility of the findings, and provide a comprehensive understanding of the research problem.

#### Operational definitions

- **Weeks of the study period**: The study includes 183 weeks, considering the WHO reporting weeks, from the 11 WHO epi-week 2020 and ends 36 WHO epi-week 2023.
- **Pre-war period**: It is the period from 11 March 2020 (the declaration of COVID-19 as a pandemic or public health emergency) until 3 November 2020 (It covers 44 WHO epi-weeks).
- **War and Total Siege**: It is the period from 4 November 2020, in which the war on Tigray started, until 5 November 2022, when the peace agreement was signed (It covers 103 WHO epi-weeks).
- **Peace agreement**: It is the period from 5 November 2022, when the peace agreement was signed, to September 2023 (It covers 44 WHO epi-weeks).
- **Positivity Rate**: This refers to proportion of confirmed cases per week relative to the total number of tests conducted during the same period.
- **Case Fatality Rate**: It is the number of deaths occurring in a given week relative to the number of confirmed cases before 2 weeks (This study considers a period of 2 weeks between confirmation of cases and deaths).

## Results

### Quantitative analysis

**Figure 1:**
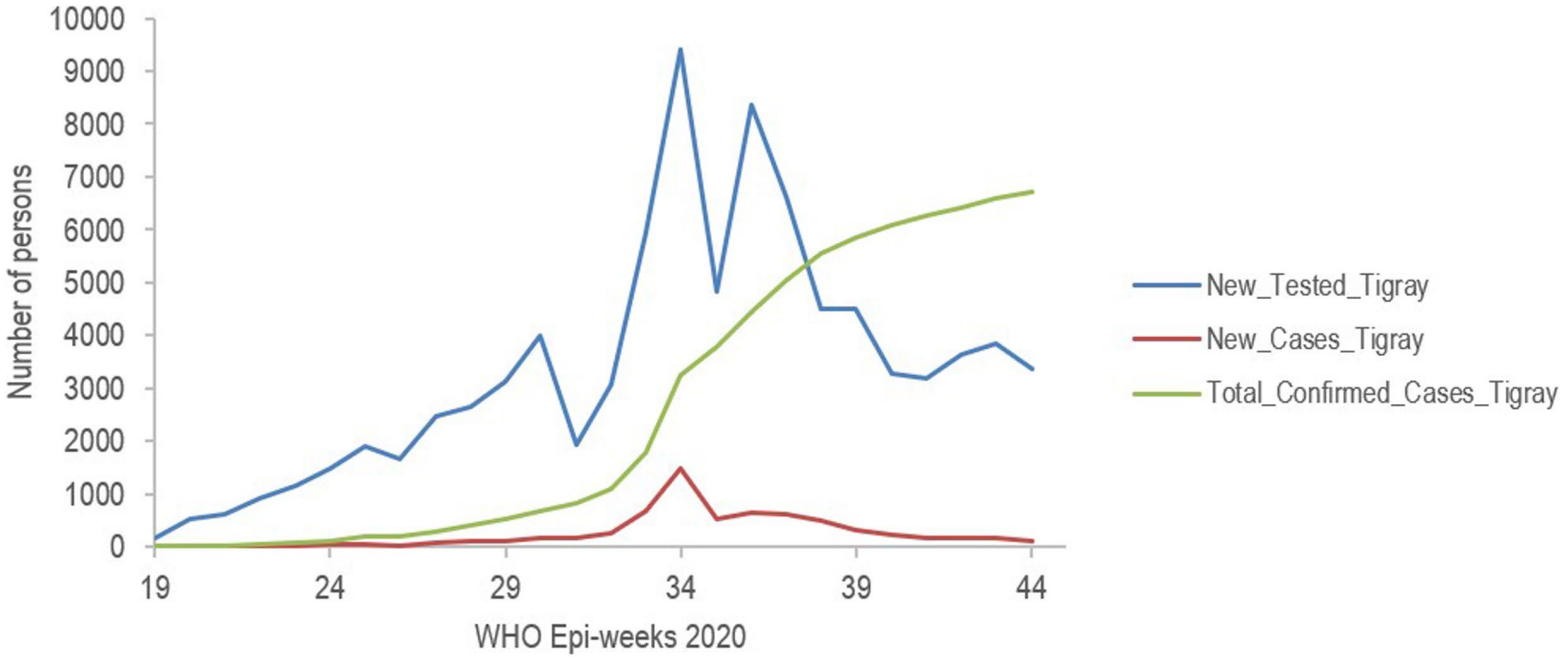
Trends in laboratory tests and test positive of COVID-19 before war in Tigray Region, Northern Ethiopia, 2020.

Tigray reported the first COVID-19 case in its territory on 25 April 2020, 6 weeks after the first case was detected in Ethiopia on 13 March 2020. Until laboratories in Tigray started their COVID-19 testing services, all the samples had been sent to Addis Ababa, where tests were verified for all other regions in Ethiopia. In Tigray, the overall number of COVID-19 tests conducted since the start of the disease until the war on Tigray was declared as 88,155, resulting in a testing rate 14 per 1,000 people. Among those tested, 6,765 individuals were confirmed to have contracted COVID-19 (Figure 1 and Table 1).

**Table 1:** Services provided in Tigray region before war, during war and total siege, and after peace agreement, 2020–2023.

Type of services	Prewar	During war and total siege	After peace agreement	Total
Total_Test	88,155	13,213	NR	101,368
Total_Confirmed_Cases	6,765	1,444	NR	8,209
Total_Death	47	85	NR	132

NR refers to Not Reported.

**Figure 2:**
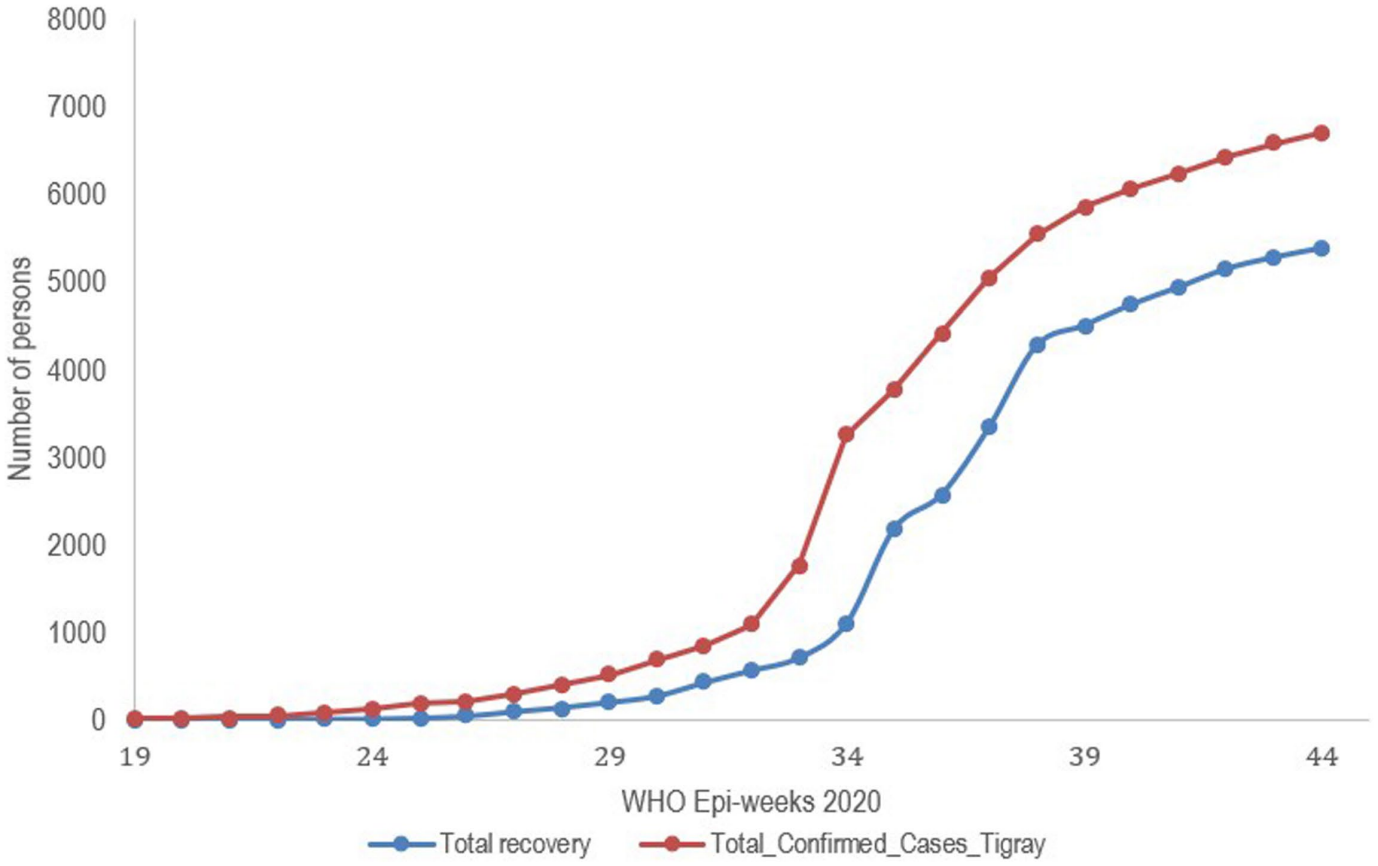
Trends of confirmed cases and recovery from COVID-19 before war in Tigray Region, Northern Ethiopia, 2020.

Cumulative cases steadily increased, reaching 6,765 just before the conflict, reflecting the spread of the virus in the population, which potentially increased the testing. Similarly, recoveries showed a parallel upward trend, with 5,388 individuals reported as recovered, highlighting ongoing medical interventions and public health efforts to manage and reduce the impact of the disease. This consistent increase in recoveries compared to cases suggested that the healthcare system was actively addressing the pandemic and successfully managing a significant number of the confirmed cases (Figures 1, 2). Unfortunately, the interruption in data recording due to the onset of the civil war did not allow us to further characterize the trend in cumulative case increase.

The positivity rate of COVID-19, that is, the percentage of all positive tests performed in the Tigray region of Northern Ethiopia during 2020, sheds light on the spread of the virus among tested individuals (Figure 1). The median positivity rate was 5.70%, indicating that half of the observed rates fell below this level. The positivity rate ranged from a minimum of 0.97% to a maximum of 15.76%, with an outlier reaching as high as 20%, suggesting time periods or locations of heightened transmission. The interquartile range (IQR) shows that the majority of positivity rates fell between 3.18 and 8.23%, illustrating a moderate level of variability in the data. The relatively high positivity rates, particularly the outlier, could indicate limited testing capacity that primarily targeted symptomatic individuals or high-risk groups, thereby potentially underestimating the true prevalence of the virus. These findings highlight the importance of increasing testing accessibility and coverage to better understand and mitigate the spread of COVID-19 in the region (Figure 1).

**Figure 3:**
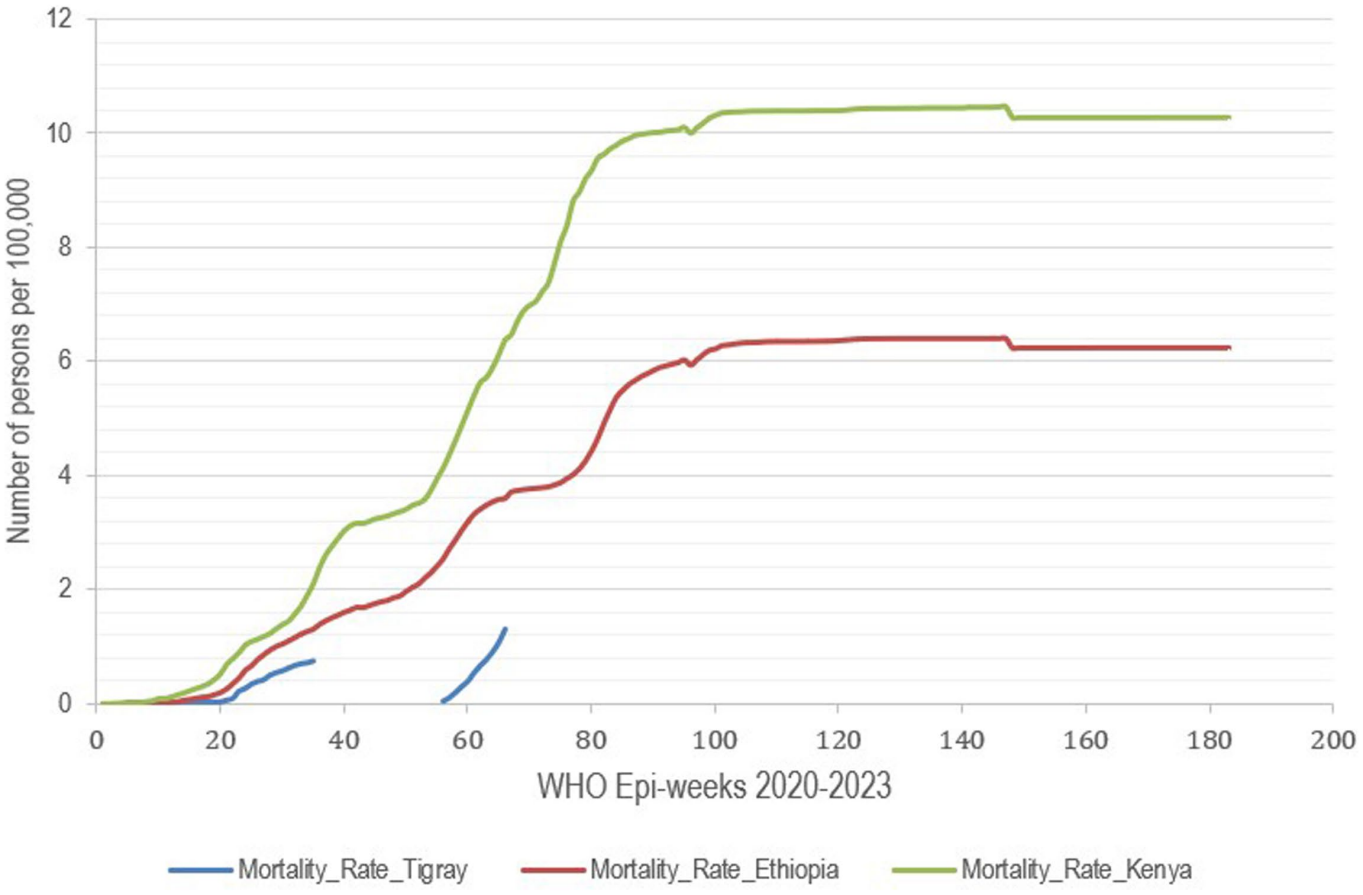
Mortality rate due to COVID-19 per 100,000 people, Tigray region, Ethiopia and Kenya, 2020–2023.

Comparing the testing and positivity rates during the war was possible for only 3 months, during which the central government remained in place until the last week of June 2021. Therefore, in the last 10 weeks before the war, among the 29,209 tests conducted, 2,232 cases were detected, which resulted in a positivity rate of 7.7%. There were 25 deaths reported. During the war, for the 10 weeks in which the services were attempted to be resumed in Mekelle city, among the 13,213 tests conducted, 3,802 cases were detected, resulting in a positivity rate of 28.8%. A total of 85 deaths were reported during this period (Figures 1, 3).

**Figure 4:**
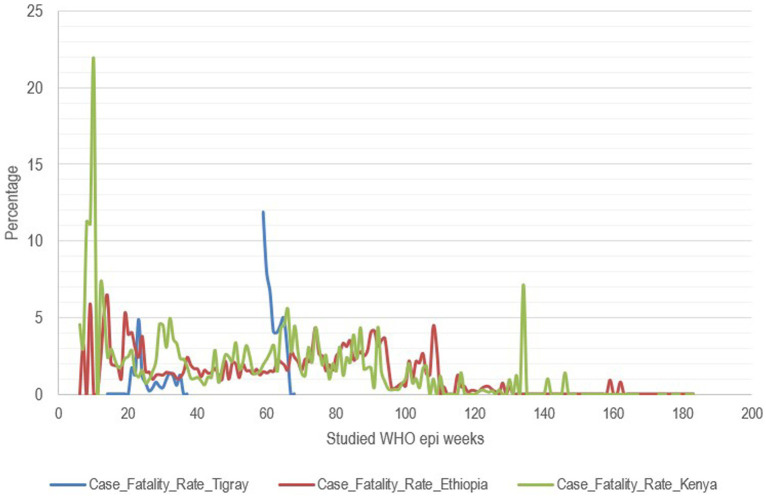
Case fatality rate of COVID-19 in percent, Tigray region, Ethiopia and Kenya, Northern Ethiopia, 2020–2023.

The case fatality rate (CFR) of COVID-19, which is the proportion of people diagnosed individuals who ultimately died from the virus in the Tigray region of Northern Ethiopia during 2020, provides crucial insights into the pandemic’s local impact. The median CFR was 1.50%, indicating that half of the reported rates were below this value. The range of CFR values varied from 0.00%, indicating time periods or locations with no deaths among confirmed cases, to a maximum of 4.85%, representing the highest observed fatality rate. The IQR reveals that the majority of CFR values fell between 0.45 and 1.19%, highlighting limited regional variability (Figure 4).

**Figure 5:**
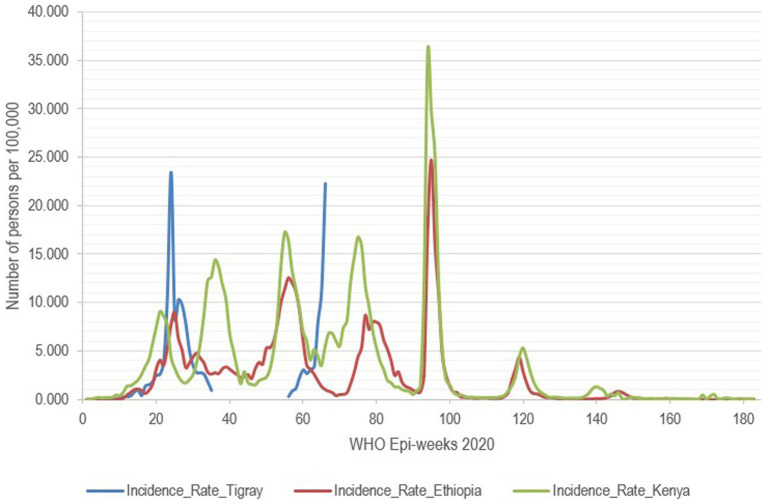
Incidence rate of COVID-19 per 100,000 people, Tigray region, Ethiopia and Kenya, Northern Ethiopia.

The incidence rate of COVID-19 in Kenya and other regions of Ethiopia showed a similar pattern, Tigray region exhibited a different trend for the available data period. Moreover, no data were available during the war and peace agreement for approximately 136 weeks, except for the 11 weeks when data were collected in the capital (Mekelle) on Tigray’s incidence, mortality, and case fatality rates (Figures 4, 5). In the first two cases, counts for the second period start again from the beginning. Hence, an analysis that extended over the whole period during which data were collected elsewhere is not possible, and only relative comparisons can be performed. Incidence and mortality rates in Tigray appear to have been increasing during the short period when data were available during the war (Figures 3, 5), while the CFR decreased during the final monitored weeks.

COVID-19 vaccination has been introduced in Ethiopia since 13 March 2021. In Tigray, vaccination was started during the interim government that was established by the federal government of Ethiopia. AstraZeneca’s first dose was provided to 118,670 people, but since there had been an ongoing war, the second dose of the vaccine was never administered to these individuals. Following the total siege, different stakeholders tried to supply COVID-19 vaccines, and 285,133 doses of Johnson & Johnson were administered. Overall, 1,992,328 individuals (45.9% of the total eligible population) were considered fully vaccinated. The majority received the one-dose vaccine, and only a few received the mix and match and booster doses. Following the Pretoria agreement, relatively better amounts and types of vaccines and medical equipment were supplied to the region. Hence, approximately 1,910,565 people were vaccinated with three types of vaccines, namely Johnson & Johnson, Pfizer, and Sinopharm (Table 2).

**Table 2:** Number of persons vaccinated by age and sex in Tigray region, Northern Ethiopia 2021–2023.

Type of vaccine	War and total seige						Peace agreement						Sum
	Age 12–17 years		Age 18–64 years		Age ≥ 65 years		Age 12–17 years		Age 18–64 years		Age ≥ 65 years		
	Female	Male	Female	Male	Female	Male	Female	Male	Female	Male	Female	Male	
AstraZeneca 1st dose*			61,258	44,359	7,832	5,221							118,670*
Sinopharm 1st dose*									27,284	25,686	1,157	918	55,045*
Sinopharm 2nd dose												33	33
Sinopharm Mix & Match									83,815	77,836	4,703	5,055	171,409
Pfizer 1st dose							142,274	157,134	14,162	12,831	1,071	108	328,552*
Pfizer booster dose							672	435	3,055	315	476	309	8,097
Pfizer Mix & Match									336	339	4	9	688
Johnson & Johnson 1st dose R1			83,815	77,836	4,703	5,055							171,409
Johnson & Johnson 1st dose R2			60,492	46,207	3,449	3,576							113,724
Johnson & Johnson 1st dose integrated with Measles									165,906	153,299	19,928	21,562	360,695
Johnson & Johnson 1st dose integrated with HPV									560,556	493,639	49,698	62,380	1,166,273
Johnson & Johnson booster dose									111,463	90,342	8,995	11,912	222,712
Sum			262,977	124,043	8,152	8,631	142,946	157,569	966,577	857,122	86,032	103,258	2,717,307

* Refers to the number of incomplete doses (only 1st dose) administered to the people.

However, among the total vaccinated people (2,314,368) during the total siege and after the peace agreement, approximately 29% were vaccinated with the first doses of Pfizer and Sinopharm, after which they had to take the second dose. Although the count is quite low, boosters and mix and match vaccinations were also provided to nearly 402,939 people. Among all the vaccinated, 53.4% were women. Concerning the age distribution, 81.1, 11.2, and 7.7% were aged 18–64 years, 12–17 years, and ≥ 65 years, respectively (Table 2).

### Qualitative analysis

The key informants have been discussing their respective divisions or departments and the region’s overall responses to COVID-19. According to the qualitative analysis, a total of 210 codes were categorized into 14 sub-themes, which were then condensed into four major themes (Table 3).

**Table 3:** Summary of themes and sub-themes used for the Qualitative Data Analysis (QDA).

Main themes	Sub-themes
1. Healthcare Resilience and Response	Case management
	IPC-wash
	Surveillance/Data management
	Laboratory
	Risk Communication and Community Engagement (RCCE)
	Logistics (Pharmaceutical and non-pharmaceutical)
	Leadership & Coordination
2. Psycho-social and economic Impact of COVID-19.	Social
	Psychological
	Economical
3. Challenges faced	Weaknesses
	Threats observed
4. Lessons learned	Strengths to be maintained
	Opportunities created

### Healthcare resilience and response

The key interviewees stated that the leadership of the regional state had taken substantial measures following the declaration of the pandemic. The Emergency Operation Centre (EOC) was constructed with seven pillars or components to coordinate the region’s comprehensive responses. To reach smaller administrative regions, each pillar was structured with a defined chain of command, and other regions viewed Tigray’s response as a model. Prior to the war, certain restrictions were put into place and adhered to in its leadership and coordination pillar, which were valued by all informants. Additionally, data management and surveillance frequently released information about events and general progress, test results, tracking contacts to cases, and other pertinent details. Laboratories were established in six major cities of the region, in line with the status of zonal administrations.

The screening process focused on persons who arrived by air and land transportation, and quarantine centers were set up around the region. Due to the stringent and well-coordinated follow-up efforts by many stakeholders, the pandemic’s impact in the region was extended by approximately 6 weeks. Prior to the cases being reported, community education was increased. The respondents said that the health system was well-organized and robust prior to the war, particularly with the presence of health extension workers and the Women’s Development Army. For 10 days, house-to-house screening was conducted in all of Tigray, identifying the primary symptoms and providing information on prevention techniques. As incidents were found, actions by each pillar were reinforced through advocacy and training. In support of this strength, one respondent stated:

*“…all sectors were working in a well-organized way. For instance, the security with its special forces was strictly following the borders and other entry points to the region to facilitate the testing. Universities, regional health bureaus, and health research institutes were working together. Besides, business owners have contributed part during this time...” IDI-3Male respondent.” [SIC]*

### Psychosocial and economic impact of COVID-19

COVID-19 has affected people’s economic, social, and psychological wellbeing in addition to the systems that operate in a particular area. People’s perceptions of the pandemic were shaped based on scientific, religious, and mixed approaches. Some people believed that COVID-19 was a result of God’s punishment for their misdeeds, while others believed that eating spicy food would protect them from infection, and garlic was regarded as a potential cure for the illness, among other beliefs that were discussed. The disease and its burden challenged health staff, who were the first to provide care. They were shunned by the community because they were believed to be the cause of the infection. People also utilized the scientific community’s definitions and potential preventive strategies. As a result, they used every mechanism to protect themselves from the disease. Each person’s mental condition was affected differently by the pandemic and its aftereffects. According to one respondent, these impacts on mental health are as follows:

*“…COVID has brought mental health crisis in the community. People have become excessively stressed and developed obsessive-compulsive disorder (OCD). As a result, especially those who have the disorder were also severely affected. Since we had a hotline service, those who quarantined were also calling us, and they feel like they were dying, and they perceived that all the severe cases were to die. However, after cases started recovering and were broadcasted through media, people started to change their perception…” KII-1 respondent.” [SIC]*

### Challenge of the pandemic

In this study, challenges are considered as internal weaknesses and threats to tackling the disease and its consequences. It is important to present the challenges faced while implementing the strategies before, during, and after the peace agreement. Similar to many other countries, the region was challenged by the characteristic features of the disease, which was also described as inducing panic. Hence, extra regulations that did not consider the real framework were adopted and implemented. The ignorance of scientific methods observed at a leadership level, such as the belief that quarantine is unnecessary when transmission occurs at the community level, had negative impacts. COVID-19 has disrupted other health programs, affecting the budget, manpower, and resources.

*“… the health workers displaced, the people afraid and dispersed along different parts, health facilities destroyed, medicines and equipment’s were damaged and looted. Consequently, the disease entered the community totally. However, people have observed more deaths and severe disabilities due to the war. Hence, they started thinking that there was another killing event (war), and finally, they ignored COVID-19. Hence, war has become one of the triggering factors for the distribution of the disease. Besides, war has destroyed the health system. It interrupted all our progress against the disease…“KII-4 Male respondent,*

The technical challenges encountered in the response were exacerbated by prewar political tensions, which ultimately led to an escalated and full-scale war. The war in Tigray has led to a total collapse of the health system in the region, including the EOS established, in which most of the health facilities were damaged and looted. Improved services, such as molecular or gene testing in laboratories, faced significant challenges, leading to a shift toward rapid diagnostic testing (RDT). However, the availability of these kits was also threatened. Not only were prevention and testing services challenged, but the case management pillars were also critically affected, especially due to the shortage of mechanical ventilators and skilled professionals to manage critical cases, resulting in loss of life. Respondents also reported that medicines were used as weapons of war. Besides, major attention was paid to other lifesaving activities related to war injuries. However, COVID-19 was considered the least priority, as it follows its natural course without any intervention, and studies on its prevalence and impact were not planned.

As the war intensified and the federal government took control of the region’s capital, it has been reported that COVID-19 responses were relaunched only in Mekelle City, where it has been safe and easy to implement again. Although it was only for a few months, the first dose of the AstraZeneca vaccine was administered. Then, some weeks later, Tigray forces took control of the region; as a result, vaccination was interrupted, and the second dose was not administered at all. Tigray had not benefited from the vaccination programs. Other vaccination campaigns were conducted without knowing the COVID-19 status of people due to a lack of resources and budget. These challenges continue to impact demand creation strategies such as advocacy and social mobilization. All difficulties, including the war, have revealed the fragility of the health system and can serve as a foundation for designing a stronger system than earlier. Experts from the EOC, along with others, explained the challenges and consequences of the war as follows:

*“…due to the war, major parts of the region haven't been accessible, the community increased the doubt on the vaccine, communication blackout happened in which we haven't communication means especially to conduct adverse event following immunization with COVID-19, there was no transport and no budget. [..] high resistance heard from the community from being vaccinated due to the war and perceived that the vaccine could be sent by the Federal government to kill them…” KII 8*

### Lessons learned

Considering the strengths and opportunities gained from overall performance in the region, respondents shared various lessons learned. Respondents acknowledged the support provided by all respected organizations and individuals, which, in the beginning, helped reduce mortality and morbidity significantly. The EOC followed and used the interim guides from the WHO and CDC to improve contact tracing, surveillance, and case management. It created a great opportunity to develop strong communication between local experts and international professionals or institutions. Key informants have emphasized the crucial role of health workers’ dedication, especially considering that they worked without being paid and in the absence of equipment. Moreover, they described that all resources, including machines that had never been utilized in the region before, were mobilized, helping to strengthen capacity and enabling the region to respond effectively to any future emergencies. To gain access and address the gap, the EOC established a liaison officer in Addis Ababa who connected the region with the available global and national resources. All in all, these lessons resulted in the system’s synergetic efforts. A key informant explained the importance of EOS and how it can be considered a lesson learned:

*“…The incident management system in Tigray was implemented in a traditional way, whereas now, we have got the opportunity to establish an Emergency Operation Center, which could also serve to manage other emergencies. It also helps to refresh our surveillance system, and strengthen our molecular testing technics and different kinds of skills also acquired by our health professionals.…” KII 7: [SIC]*

## Discussion

The war in Tigray has been one of the deadliest conflicts in the world recently: in the last 2 years, it is estimated that between 500,000 and 1,000,000 civilians have died (20), over 2 million people have been internally displaced, over 55,000 refugees have fled to Sudan, and the population of the regional state has been directly affected by the conflict, with nearly 5.2 million people requiring humanitarian aid (21–24). The region has become exposed to the pandemic, and the combined effects of the war have significantly exacerbated the public health catastrophe.

Before the war began, the Tigray region experienced an increasing trend in both COVID-19 cases and recoveries, indicating not only a growing outbreak but also corresponding efforts to manage and treat the virus effectively (Figure 2). Globally, the World Health Organization (WHO) recommended keeping the positivity rate below 5% as a benchmark for adequate testing and control of the pandemic. The median positivity rate in Tigray exceeded this benchmark, aligning more closely with the higher positivity rates observed in regions with limited testing capacity or significant transmission. The relatively low case fatality rate (CFR) compared to the global average in 2020, which ranged between 1 and 3%, suggests possible factors such as effective healthcare management, a predominantly younger population demographic, or the potential underreporting of deaths or cases.

The WHO defined an attack on healthcare as any verbal or physical act of violence, obstruction, or threat that interferes with the availability, access, and delivery of services (25). According to the qualitative findings, in Tigray, there have been deliberate attacks on health facilities and the medical resources that entered into the region. This was also supported by different reports, including reports from the WHO, which have shown that health facilities across the Tigray region have been looted, vandalized, and destroyed in a deliberate and widespread attack on healthcare. Of 106 health facilities visited by MSF teams between mid-December and early March, nearly 70% had been looted, and more than 30% had been damaged; only 13% functioned normally (26, 27). Similarly, a study from Ukraine showed that in territories occupied by Russian troops, medical facilities were largely non-functional, and there were no laboratory diagnostics, PCR tests, or rapid tests conducted (6).

Quantitative data demonstrated that only during 10 weeks, and only in one city (Mekelle), out of the 103 WHO epi-weeks coinciding with the war and the complete siege, there was an opportunity to manage patients and administer tests RDT tests. The key informants have justified that such limitations could be ascribed to the damaged healthcare infrastructure, interrupted supply chains, insecurity, and overburdened health systems. Managing COVID-19 during this time presents substantial hurdles. Personnel, PPE, and kit shortages hindered testing and monitoring. Furthermore, COVID-19 was frequently given the least amount of attention since urgent survival requirements, including food, medical attention for gunshot wounds, insecurity, electricity, and water supplies, took precedence first. This problem has also been illustrated in the health cluster report, which notes that in the majority of conflict-affected areas, COVID-19 response efforts have been inadequate or non-existent (28).

Vaccines provide strong protection, but acquiring that protection requires both time and the completion of necessary doses. People should receive all the required vaccine doses to achieve full immunity. Different studies have recommended that the population be informed about the importance of complete vaccination, especially as variants of concern become dominant in many countries (29–31). When two-dose vaccines are employed, only partial protection is achieved after the first dose, and the second dose increases that protection (31). A study estimates the high effectiveness of the BNT162b2 vaccine in preventing symptomatic COVID-19 in a non-controlled setting, similar to the vaccine efficacy reported in the randomized trial. A study from England has estimated that vaccine effectiveness during the follow-up period starting 7 days after the second dose was 92% for documented infection, 94% for symptomatic COVID-19, 87% for hospitalization, and 92% for severe COVID-19 (32). Another study from California, US, demonstrated that vaccination effectiveness was 91.3% (95%CI: 249 79.7–96.3%) at ≥15 days after the second dose for the symptomatic infection (33).

However, some studies show a substantial correlation between previous COVID-19 and vaccine side effects. Moreover, the degree of side effects varied among different vaccine brands (34), and there were significant differences between immunizations in terms of menstrual disruptions (35). Since war-affected and conflict-affected nations create particularly favorable circumstances for the virus’s ongoing spread, focusing on immunization efforts could also serve as a successful pandemic prevention strategy (36). Following the fourth national campaign in May 2023, the Ethiopian Federal Ministry of Health launched follow-up mini-campaigns to reach conflict-affected areas (37). In Tigray, the vaccinations were administered without any prior COVID-19 status. Additionally, no adverse events following immunization (AEFI) studies were conducted, and individuals lacked the necessary communication tools to report the vaccines’ acute negative effects.

Only 45.9% of the eligible population was fully vaccinated, while approximately 502,267 individuals (29%) had received only one of the two doses required. This situation largely stems from missed immunization campaigns in Tigray. This coverage places the region below Nigeria, where 71.6% of eligible individuals had received all recommended vaccinations by 31 December 2023 (38), but above the Democratic Republic of the Congo, where 16% of the population had completed the primary COVID-19 vaccination series (39). Given Mekelle’s high positivity rate and the region’s presumed high level of mobility, the frequency and complications of COVID-19 infections may have been exacerbated. In this regard, key informants reported that they were not able to treat adverse events following COVID-19 immunization due to various reasons. This finding is also in line with research indicating that the conflict in Ukraine significantly worsened the impact of COVID-19 (6).

Health information systems, especially civil registration systems that document events and causes of death, frequently stop working in war-affected populations, posing a significant barrier to measuring the health impacts of conflict (15). Qualitative data also revealed that the health information system, as a core component of Tigray’s health infrastructure, had completely collapsed, making it impossible to obtain any data, including on COVID-19 cases and fatalities. This breakdown suggests that the extent of the pandemic’s spread in the region may be far greater than what the available data indicate.

### Strengths and limitations of the study

The study attempted to triangulate data from various databases to improve overall data quality in a context where, as in many other developing countries, the health information system faces challenges related to data completeness, consistency, and timeliness. Due to the protracted nature of the war, the research was unable to estimate long-term trends of the disease, limiting access to comprehensive data on COVID-19 testing, management, and mortality. On the other hand, since the KII was conducted in December 2024, recall bias may have affected participants’ ability to remember specific tasks and the associated challenges. To mitigate potential bias, key informants were asked cross-checking questions to ensure the reliability and consistency of their responses.

## Conclusion

While the region initially succeeded in delaying the onset of the disease and maintaining lower positivity and fatality rates, the war in Tigray led to severe disruption and eventual collapse of the healthcare system. As a result, COVID-19 was largely neglected and deprioritized, even after the signing of the cessation of hostility agreement. Thus, restoring damaged healthcare infrastructure is essential. Efforts should include expanding decentralized healthcare delivery, such as mobile clinics and telemedicine, strengthening public health measures, such as disease surveillance, and ensuring the availability of essential supplies. In addition to capacity building of healthcare workers, integrating mental health services into primary care should be considered. Further studies are also recommended to investigate long-term side effects of COVID-19, adverse effects following booster doses, and differences in side effect profiles between heterologous and homologous vaccination regimens. These insights are crucial for improving the management of high-risk populations and patients experiencing vaccine-related side effects.

## Data Availability

The raw data cannot be freely shared due to restrictions imposed by the Ethical Committees, which do not allow the open/public sharing of individual-level data. However, aggregated data are accessible to other researchers upon request. Please ask the corresponding author.

## References

[1] World Health Organization. Novel coronavirus (2019-nCoV). Report No.: SITUATION REPORT-1. (2020). Available online at: https://www.who.int/docs/default-source/coronaviruse/situation-reports/20200121-sitrep-1-2019-ncov.pdf

[2] WHO Director-General’s opening remarks at the media briefing on COVID-19 – 11th March 2020. (2023). Available online at: https://www.who.int/director-general/speeches/detail/who-director-general-s-opening-remarks-at-the-media-briefing-on-covid-19---11-march-2020

[3] World Health Organization. Considerations for implementing and adjusting public health and social measures in the context of COVID-19: Interim guidance. (2023). Available online at: https://www.who.int/publications/i/item/considerations-in-adjusting-public-health-and-social-measures-in-the-context-of-covid-19-interim-guidance

[4] HelpAge International. Seven principles for a rights-based public health response to COVID-19. (2020). Available online at: https://www.helpage.org/silo/files/seven-principles-for-a-rights-based-public-health-response-to-covid19.pdf

[5] WHO coronavirus (COVID-19) dashboard. Available online at: https://covid19.who.int/

[6] Chumachenko D Chumachenko T. Impact of war on the dynamics of COVID-19 in Ukraine. BMJ Glob Health. (2022) 7:e009173. doi: 10.1136/bmjgh-2022-009173, PMID: 35428677 PMC9013784

[7] Hemmeda L Shabani MM Kolawole BO Muhammad SA Fatima K Siddiqui A. Third wave of COVID-19 pandemic in Africa: challenges and recommendations. Ann Med Surgery. (2022) 80:80. doi: 10.1016/j.amsu.2022.104314, PMID: 35945972 PMC9352644

[8] Tagoe ET Sheikh N Morton A Nonvignon J Sarker AR Williams L. COVID-19 vaccination in lower-middle income countries: National Stakeholder Views on challenges, barriers, and potential solutions. Front Public Health. (2021) 9:709127. doi: 10.3389/fpubh.2021.709127 34422750 PMC8377669

[9] Kunyenje CA Chirwa GC Mboma SM Ng’ambi W Mnjowe E Nkhoma D. COVID-19 vaccine inequity in African low-income countries. Front Public Health. (2023) 11:1087662. doi: 10.3389/fpubh.2023.1087662 36950103 PMC10025287

[10] Impact of COVID-19 on people’s livelihoods, their health and our food systems. Available online at: https://www.who.int/news/item/13-10-2020-impact-of-covid-19-on-people’s-livelihoods-their-health-and-our-food-systems

[11] Lee J. Mental health effects of school closures during COVID-19. Lancet Child Adolescent Health. (2020) 4:421. doi: 10.1016/S2352-4642(20)30109-7, PMID: 32302537 PMC7156240

[12] Bwire G Ario AR Eyu P Ocom F Wamala JF Kusi KA. The COVID-19 pandemic in the African continent. BMC Med. (2022) 20:167. doi: 10.1186/s12916-022-02367-4, PMID: 35501853 PMC9059455

[13] Nicola M Alsafi Z Sohrabi C Kerwan A Al-Jabir A Iosifidis C. The socio-economic implications of the coronavirus pandemic (COVID-19): a review. Int J Surgery. (2020) 78:185–93. doi: 10.1016/j.ijsu.2020.04.018, PMID: 32305533 PMC7162753

[14] Jessica A. B Jennifer B. N. Global health security index: advancing collective action and accountability amid global crisis. (2021). Available online at: www.GHSIndex.org

[15] Murray CJL. Armed conflict as a public health problem. BMJ. (2002) 324:346–9. doi: 10.1136/bmj.324.7333.346, PMID: 11834565 PMC1122272

[16] Choudhary OP Saied AA Priyanka ARK Maulud SQ. Russo-Ukrainian war: an unexpected event during the COVID-19 pandemic. Travel Med Infect Dis. (2022) 48:102346. doi: 10.1016/j.tmaid.2022.102346, PMID: 35487342 PMC9042412

[17] The International Monetary Fund (IMF). CRISIS UPON CRISIS. Report No.: imf–annual–report–2022–english.pdf. (2022). Available online at: https://www.imf.org/external/pubs/ft/ar/2022/downloads/imf-annual-report-2022-english.pdf

[18] Gebreeyesus M. Ethiopia’s response to the COVID-19 pandemic: measures, impacts and lessons. (2022). Available online at: https://unctad.org/system/files/official-document/BRI-Project_RP25_en.pdf

[19] Tigray Health Bureau. Tigray health sector annual bulletin 2021. (2022). Available online at: https://tigrayeao.info/wp-content/uploads/2022/02/final-annual-bulletin-compressed.pdf

[20] Endale A. Conflict pushes 3M deeper into poverity. Reporter. (2023). Available online at: https://www.thereporterethiopia.com/34088/

[21] Borrell Josep. We need humanitarian access to Tigray as urgent first step towards peace in Ethiopia. European Union External Action. (2021). Available online at: https://www.cidob.org/en/publications/scramble-external-powers-and-local-agency-horn-africa

[22] Ylönen A. A scramble of external powers and local agency in the horn of Africa. NotesInt. (2022) 280:1–7.

[23] World Health Organization. Crisis in Northern Ethiopia. (2023). Available online at: https://www.who.int/emergencies/situations/crisis-in-tigray-ethiopia

[24] Gesesew H Berhane K Siraj ES Siraj D Gebregziabher M Gebre YG. The impact of war on the health system of the Tigray region in Ethiopia: an assessment. BMJ Glob Health. (2021) 6:e007328. doi: 10.1136/bmjgh-2021-007328, PMID: 34815244 PMC8611430

[25] World Health Organization. Attacks on health care in the context of COVID-19.pdf. (2020). Available online at: https://www.who.int/news-room/feature-stories/detail/attacks-on-health-care-in-the-context-of-covid-19

[26] Medecins Sans Frotieres. Health facilities targeted in Tigray. (2021). Available online at: https://www.doctorswithoutborders.ca/health-facilities-targeted-in-tigray/

[27] Tigray the deliberate destruction of a health system.pdf. Available online at: https://www.devex.com/news/tigray-the-deliberate-destruction-of-a-health-system-102252

[28] Northern-Ethiopia-health-cluster-bulletin. (2021). Available online at: https://healthcluster.who.int/publications/m/item/ethiopia-health-cluster-bulletin-october-2021

[29] European Centre for Disease Prevention and Control. Partial COVID-19 vaccination, vaccination following SARS-CoV-2 infection and heterologous vaccination schedule. (2021). Available online at: https://www.ecdc.europa.eu/sites/default/files/documents/Partial%20COVID%20vaccination%20and%20heterologous%20vacc%20schedule%20-%2022%20July%202021.pdf

[30] Haas EJ Angulo FJ McLaughlin JM Anis E Singer SR Khan F. Impact and effectiveness of mRNA BNT162b2 vaccine against SARS-CoV-2 infections and COVID-19 cases, hospitalisations, and deaths following a nationwide vaccination campaign in Israel: an observational study using national surveillance data. Lancet. (2021) 397:1819–29. doi: 10.1016/S0140-6736(21)00947-8, PMID: 33964222 PMC8099315

[31] World Health Organization. Vaccine efficacy, effectiveness and protection. (2021). Available online at: https://www.who.int/news-room/feature-stories/detail/vaccine-efficacy-effectiveness-and-protection

[32] Dagan N Barda N Kepten E Miron O Perchik S Katz MA. BNT162b2 mRNA COVID-19 vaccine in a Nationwide mass vaccination setting. N Engl J Med. (2021) 384:1412–23. doi: 10.1056/NEJMoa2101765, PMID: 33626250 PMC7944975

[33] Andrejko KL Pry J Myers JF Jewell NP Openshaw J Watt J. Prevention of COVID-19 by mRNA-based vaccines within the general population of California. (2021). doi: 10.1093/cid/ciab640 PMC8406879 34282839

[34] Beatty AL Peyser ND Butcher XE Cocohoba JM Lin F Olgin JE. Analysis of COVID-19 vaccine type and adverse effects following vaccination. JAMA Netw Open. (2021) 4:e2140364. doi: 10.1001/jamanetworkopen.2021.40364, PMID: 34935921 PMC8696570

[35] Abdollahi A Naseh I Kalroozi F Kazemi-Galougahi MH Nezamzadeh M Sabeti Billandi S. Comparison of side effects of COVID-19 vaccines: Sinopharm, AstraZeneca, sputnik V and Covaxin in women in terms of menstruation disturbances, hirsutism and metrorrhagia: a descriptive-analytical cross-sectional study. Int J fertil Steril. (2022) 16:237–43. doi: 10.22074/ijfs.2022.544706.1236, PMID: 36029063 PMC9396006

[36] Gugushvili A Mckee M. The COVID-19 pandemic and war. Scand J Public Health. (2022) 50:16–8. doi: 10.1177/1403494821993732, PMID: 33612033 PMC8807542

[37] COVID-19 vaccination integration assessment Ethiopia case study. (2024). Available online at: https://usaidmomentum.org/wp-content/uploads/2024/04/Ethiopia_COVID-19_Integration_Assessment508.pdf

[38] Arlington VA. MOMENTUM routine immunization transformation and equity. COVID-19 vaccination program in review. Nigeria: JSI Research & Training Institute, Inc (2024).

[39] Ndjadi Y. Routine immunization transformation and equity; COVID-19 vaccination program in review – Democratic Republic of Congo. (2024). Available online at: https://usaidmomentum.org/wp-content/uploads/2024/06/DRC-COVID-19-Vaccination-Program-in-Review_508.pdf

