# Supplementary material for: COVID-19 pandemic during the war in Tigray, Northern Ethiopia: a sequential mixed-methods approach
Source: Front Public Health. 2025 Apr 16;13:1553452. doi: 10.3389/fpubh.2025.1553452 (PMC12040842; doi:10.3389/fpubh.2025.1553452)
Supplement: Supplementary file 1 [file Data_Sheet_1.pdf]

Annex 1: Quantitative data extraction table:

Year	WHO -Epi-Week	EPHI Bulletin number	New Tested	Total Test	New Cases	Total Confirmed Cases	New recovery	Total Recovery	New death	Total Death	New Vaccination	Total Vaccination	Population	Incidence Rate	Prevalence Rate	Mortality Rate	Case Fatality Rate
