# Supplementary material for: COVID-19 pandemic during the war in Tigray, Northern Ethiopia: a sequential mixed-methods approach
Source: Front Public Health. 2025 Apr 16;13:1553452. doi: 10.3389/fpubh.2025.1553452 (PMC12040842; doi:10.3389/fpubh.2025.1553452)
Supplement: Supplementary file 2 [file Data_Sheet_2.pdf]

Annex 2: Points of discussion for Key Informant Interview (KII)

1. After the declaration of COVID-19 as Pandemic, how would you describe the overall preparations and implementations of interventions against the disease?
    1. ኮቪድ ፓንደሚክ ተባሂሉ ካብ ዝፅዋዕ: ምድላዋትን ትግበራን ምክልኻልን ምሕካምን እቲ ሕማም ከመይ ነይሩ?
2. How were the medical supplies useful to the prevention, testing and case management? What can you say about the health system in order to tackle it?
    2. ንጥፈታትን ኣቕርቦትን ምክልኻል: ናሙና: ሕክምና ከመይ ነይሮም: ብፍላይ ድማ ምስቲ ዝነብረ ስርዓተ ጥዕና ከመይ ተዋዲዱ ነይሩ?
3. What were the strengths, weaknesses, opportunities and threats observed during the preparation and implementation of activities?
    3. ዝነበሩ ጥንካረታት: ድኽመታት: ዕድላትን ማሕለኻታት እንታይ እዮም
4. After the war started, what happened to interventions? What can you say about the interaction of the events?
    4. ኮቪድን ጦርነትን ከ ብኸመይ ትገልፆ?
5. What were the changes appeared after the peace agreement?
    5. ድሕሪ ውዕሊ ፕሪቶርያ: ዝተረኸቡ ዕድላት: እንታይ እዮም
6. What are the relevances and challenges with the COVID-19 vaccination
    6. ክታበትን ወፍርታት ክታበትን: ዝነበሩ ረብሓን ፀገማትን እንታይ እዮም
